# Dietary Glycemic Load and Plasma Amyloid-β Biomarkers of Alzheimer’s Disease

**DOI:** 10.3390/nu14122485

**Published:** 2022-06-15

**Authors:** Mélissa Gentreau, Michel Raymond, Cécilia Samieri, Virginie Chuy, Catherine Féart, Claire Berticat, Sylvaine Artero

**Affiliations:** 1 Institute of Functional Genomics (IGF), University of Montpellier, CNRS, INSERM, F-34094 Montpellier, France; 2 ISEM, University of Montpellier, CNRS, EPHE, IRD, F-34095 Montpellier, France; michel.raymond@umontpellier.fr (M.R.); claire.berticat@umontpellier.fr (C.B.); 3 Bordeaux Population Health Research Center, University of Bordeaux, INSERM, BPH, UMR 1219, F-33000 Bordeaux, France; cecilia.samieri@u-bordeaux.fr (C.S.); virginie.chuy@u-bordeaux.fr (V.C.); catherine.feart-couret@u-bordeaux.fr (C.F.); 4 CHU de Bordeaux, Pôle de Médecine et Chirurgie Bucco-Dentaire, F-33000 Bordeaux, France

**Keywords:** glycemic load, refined carbohydrate, amyloid-β, Alzheimer’s disease, biomarker, cohort

## Abstract

Previous studies have highlighted links between a high-glycemic-load (GL) diet and Alzheimer’s disease in apolipoprotein E ε4 (APOE4) carriers. However, the impact of high-GL diet on plasma amyloid-β (Aβ), an Alzheimer’s disease hallmark that can be detected decades before clinical symptomatology, is unknown. This study examined the association between plasma Aβ peptides (Aβ_40_, Aβ_42_ concentration and Aβ_42_/Aβ_40_ ratio) and GL. The influence of the GL of four meal types (breakfast, lunch, afternoon snack, and dinner) was also determined. From the prospective Three-City study, 377 participants with plasma Aβ measurements, and who completed the Food Frequency Questionnaire, were selected. The association between plasma Aβ and GL was tested using an adjusted linear regression model. Lunch GL was associated with a lower plasma Aβ_42_ concentration (β = −2.2 [CI = −4.27, −0.12], *p* = 0.038) and lower Aβ_42_/Aβ_40_ ratio (β = −0.009 [CI = −0.0172, −0.0007], *p* = 0.034) in the model adjusted for center, age, sex, education level, APOE4 status, energy intake, serum creatinine, total cholesterol, and Mediterranean-like diet. No significant association was found with the GL of the other meal types. These results suggest that dietary GL may independently modulate the plasma Aβ of the APOE4 status. The mechanism underlying diet, metabolic response, and Aβ peptide regulation must be elucidated.

## 1. Introduction

Alzheimer’s disease (AD), the most common form of dementia, is a neurodegenerative disease characterized by amyloid-β (Aβ) deposition and neurofibrillary tangles. Dementia affects 50 million people worldwide and represents the main cause of dependency and disability in older adults [1]. To date, no curative pharmacological treatment is available for dementia. Therefore, it is essential to develop effective and widely applicable strategies to prevent or delay AD onset. Accumulating evidence indicates that diet interventions could be one of these strategies [2,3]. Indeed, a refined carbohydrate-rich diet is known to increase Aβ in mice brains [4,5]. Recently, a meal intervention study suggested that the Western diet, rich in saturated fat and refined carbohydrates, could exacerbate Aβ peptide concentration in plasma [6]. Importantly, the plasma concentration of Aβ_42_ and Aβ_40_ peptides may reflect Aβ burden in the brain [7]. Moreover, a high plasma Aβ_40_ concentration, low plasma Aβ_42_ concentration, and low Aβ_42_/Aβ_40_ ratio have been linked to a higher dementia risk [8,9]. However, no study in the general population has specifically examined the association between a refined carbohydrate diet and plasma Aβ biomarkers. Thus, the investigation of this association may provide evidence that a refined carbohydrate diet is an environmental factor that promotes AD pathogenesis via plasma Aβ. Additionally, this would open the possibility of acting on this modifiable dietary factor to reduce the prevalence of dementia in the general population. In this context, our previous work emphasized that a high-glycemic-load (GL) diet increases the risk of dementia in apolipoprotein E ε4 (APOE4) carriers, the main genetic risk factor of AD [10]. In addition, an interventional study demonstrated that diet may change the plasma concentration of Aβ peptides according to the APOE4 carrier status [6,11], suggesting that APOE4 status could be an effect modifier. Moreover, our work highlighted the stronger impact of the GL of an afternoon snack (the meal with the highest content in refined carbohydrates) on the risk of dementia [10], suggesting a risk variability according to the meal type. Indeed, snacks and breakfast tend to be richer in refined carbohydrates and therefore have higher GL values than the main meals (i.e., lunch and dinner) [12].

Based on these findings, we hypothesized that a high-GL diet might be associated with plasma Aβ peptide levels. Therefore, we used data from the prospective Three-City (3C) cohort study to examine the association between GL and plasma Aβ_40_ and Aβ_42_ concentration, as well as Aβ_42_/Aβ_40_ ratio. We also evaluated the potential interactive effect of APOE4 carrier status and the effect of the daily, breakfast, lunch, afternoon snack, and dinner GL values on this association.

## 2. Materials and Methods

### 2.1. Study Sample

**Figure 1:**
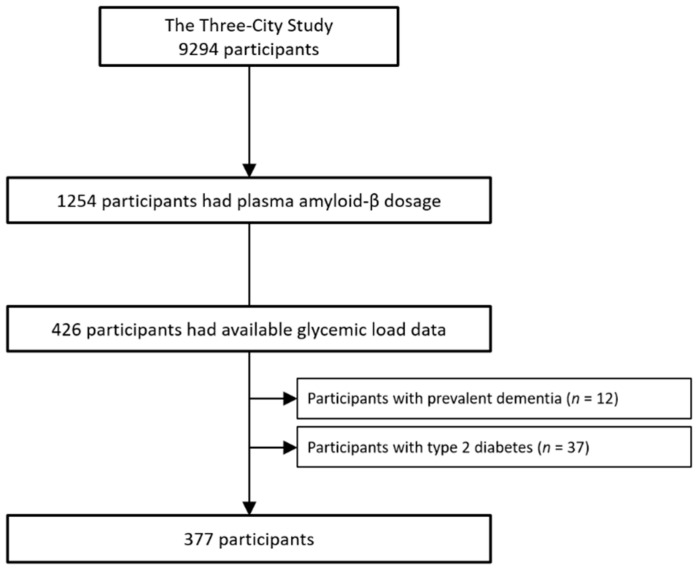
Flow chart.

The 3C study is a population-based, prospective study on the relationship between vascular factors and dementia. The study started in 1999–2000 and included participants, aged 65 years or older, from three French cities: Bordeaux, Dijon, and Montpellier [13]. The Consultative Committee for the Protection of Persons Participating in Biomedical Research at Kremlin-Bicêtre University Hospital (Paris, France) approved the 3C Study protocol, and all participants gave their written informed consent. Baseline data collection included sociodemographic, clinical, and lifestyle characteristics, medication use, neuropsychological testing, and clinical examination with blood sampling. At baseline, 1254 participants were randomly selected from the three centers, stratified by center, sex, and age, for plasma Aβ concentration measurement [9]. From this subsample, participants at the Bordeaux and Montpellier centers who completed a 148-item Food Frequency Questionnaire (FFQ) at 2-year and 4-year follow-up visits, respectively, (Figure 1) were selected.

### 2.2. Dietary Data

The 148-item semi-quantitative FFQ was divided into breakfast, lunch, dinner, and snacks between meals. The GL is an indicator of the cumulative exposure to postprandial glycemia and reflects insulin demand, induced by the carbohydrate intake [14,15]. GL values were obtained from the International Table of Glycemic Index [16] and internet updates (www.glycemicindex.com (accessed on 11 June 2019)). GL items are defined as “low” when the GL value is below 10 and as “high” when the value is above 20 [17]. As previously described, GL was calculated for breakfast, lunch, afternoon snack (“goûter” in French, corresponding to a snack between lunch and dinner), dinner, and the entire day, and validated with the GL values from the 24 h dietary recall [10]. GL values were within the same range of variation as those from another study on a French population (Table S1) [18]. In the Bordeaux 3C study, carbohydrate intake did not seem to change during the follow-up [19]. Thus, it was hypothesized that the GL did not change from baseline until the visit when the FFQ was completed. Energy intake and adherence to a Mediterranean-like diet were also determined [10]. Diet quality, for example, from a Mediterranean diet, may influence the plasma concentration of Aβ peptides [20] as well as the GL values [21,22]. The Mediterranean-like diet score was categorized as low (0–3), moderate (4–5), and high (6–9). Missing values of the Mediterranean-like diet score (8%) were added in a fourth category.

### 2.3. Plasma Amyloid-β Peptide Assessment

The plasma Aβ peptide assay is described elsewhere [9]. Briefly, blood samples were collected in tubes containing sodium EDTA. After centrifugation, samples were aliquoted and stored at −80 °C. Plasma Aβ peptides were measured using the INNO-BIA Kit (Innogenetics, Ghent, Belgium) based on the multiplex xMAP technique (Luminex, Austin, TX, USA). Aβ_40_ and Aβ_42_ concentrations were expressed in pg/mL, and the Aβ_42_/Aβ_40_ ratio was calculated.

### 2.4. Incident Dementia Diagnosis

At the 2-, 4-, 7-, 10-, 12- and 15-year follow-up visits, a neurologist examined participants with suspected dementia on the basis of their neuropsychological test scores. Then, an independent committee of neurologists evaluated all potential cases of dementia to reach a consensus on the diagnosis and etiology based on the Diagnostic and Statistical Manual of Mental Disorders, Fourth Edition [23].

### 2.5. Other Covariates

Education level was defined as no school, primary school, high school, or graduate level. APOE genotyping was performed as previously described [24]. APOE4 carriers were defined as carrying at least one *ε*4 allele. Diabetes was defined as treated diabetes, fasting glycemia > 7 mmol/L, and self-reported diabetes. Serum creatinine (mmol/L) was measured with the Jaffe method [25]. Total cholesterol (mmol/L) was quantified using routine enzymatic methods. Serum creatinine and total cholesterol are biological factors that may influence the plasma concentration of Aβ peptides [26,27].

### 2.6. Statistical Analyses

Linear regression models were used to evaluate the association between GL and Aβ peptide concentration/ratio. The models were first adjusted for energy intake. Then, multicollinearity was tested using the variance inflation factor [28]. As GL showed a high variance inflation factor value when energy intake was added to the models, the residual method was used with all GL values [14,29]. The first model was adjusted for center, age, sex, education level, APOE4 status, and energy intake. The second model was additionally adjusted for serum creatinine, total cholesterol, and Mediterranean-like diet, which may influence the plasma concentration of Aβ peptides [20,26,27]. The interaction with APOE4 carrier status was also tested. The model assumptions were checked, and outliers were removed (*n* = 4 for Aβ_40_, *n* = 3 for Aβ_42_, and *n* = 2 for Aβ_42_/Aβ_40_) by looking at the graphs and using the Bonferroni method with the ‘car’ package in R [30]. For the sensitivity analysis, participants with incident dementia were excluded to avoid potential reverse causation. All statistical analyses were performed using R version 3.6.1(R Core Team, Vienna, Austria). Two-sided Fisher tests were used, and a *p* value < 0.05 was considered statistically significant.

## 3. Results

At FFQ completion, the mean age of the 377 participants was 76.1 (±5.2) years and 60.2% were women (Table 1). Participants in the high tertile group of the daily GL included fewer women, and these participants tended to have a higher Mediterranean-like diet score compared with the low tertile group.

To evaluate the association between GL and plasma Aβ peptide concentration and ratio according to APOE4 status, the GL × APOE4 interaction was tested. As no significant interaction was detected between GL and APOE4 status, the results are presented for the whole sample.

**Figure 2:**
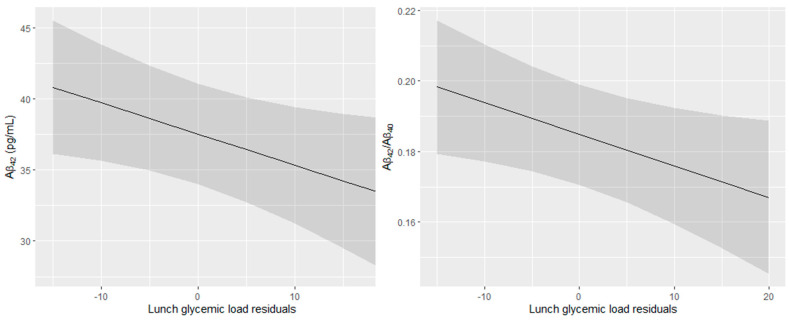
Predicted Aβ_42_ concentration and Aβ_42_/Aβ_40_ ratio in plasma in function of the lunch glycemic load residuals (95% confidence intervals). All models were adjusted for center, age, sex, education level, APOE4 status, energy intake, serum creatinine, total cholesterol, and Mediterranean-like diet.

After removing outliers, no significant association between daily GL and plasma Aβ peptide concentration and ratio was detected in both models (Table 2). In model 1, breakfast GL tended to be associated with higher Aβ_40,_ but not after additional adjustments in model 2. Lunch GL tended to be associated with lower Aβ_42_ in model 1, and the association between lunch GL and lower Aβ_42_ and ratio became significant after the adjustment for serum creatinine, total cholesterol, and a Mediterranean-like diet (model 2). The predicted Aβ_42_ and Aβ_42_/Aβ_40_ ratio values as a function of the lunch GL are presented in Figure 2. Finally, in both models, afternoon-snack and dinner GL were not associated with plasma Aβ peptide concentrations and ratio.

Participants who developed dementia during the 15-year follow-up (*n* = 51) were older than those who remained dementia-free. They were more likely to be women and APOE4 carriers. They also had a lower education level and a lower Mediterranean-like diet score than dementia-free participants (Table S2). In the sensitivity analysis that evaluated the association between GL and Aβ, after exclusion of the participants with incident dementia, the results were similar to those of the main analysis. The associations between lunch GL and plasma Aβ_42_ concentration and ratio became stronger (Table S3).

These results showed that lunch GL was associated with lower plasma Aβ_42_ concentration and lower Aβ_42_/Aβ_40_ ratio for a 10-point increase in the GL value per day, independently of the APOE4 carrier status.

## 4. Discussion

This study highlights the links between dietary GL and plasma Aβ biomarkers independently of the APOE4 carrier status. We found that lunch GL was associated with a lower plasma Aβ_42_ concentration and lower Aβ_42_/Aβ_40_ ratio. These associations remained significant after adjustment for center, age, sex, education level, APOE4 status, energy intake, serum creatinine, total cholesterol, and Mediterranean-like diet. Despite the cross-sectional design of our study, these findings suggest that dietary GL, as a proxy of refined carbohydrate consumption, may negatively modulate the plasma Aβ biomarkers. Indeed, lower plasma Aβ_42_ and lower Aβ_42_/Aβ_40_ ratio have been associated with higher risk of dementia in longitudinal studies [8,9]. Nevertheless, since no association was found between other meal types and plasma Aβ biomarkers, this study should be interpreted with caution and replicated in longitudinal studies with larger sample sizes.

Studies on the relationship between refined carbohydrate consumption and Aβ are scarce. Only two interventional studies investigated the impact of a refined carbohydrate-rich diet on Aβ in plasma and cerebrospinal fluid. However, these studies combined a diet rich in refined carbohydrates and in saturated fat, and thus could not highlight the specific effect of refined carbohydrates [6,31]. One neuroimaging study showed that high-GL diet was associated with global and regional Aβ burden in the brain of cognitively normal subjects [32]. Indeed, a high-GL diet was associated with higher Aβ burden in the anterior and posterior cingulate gyrus, the lateral temporal lobe, the precuneus, and the superior parietal lobe.

Previous studies on the relationship between dietary GL and Aβ never considered the different meal types. Our results highlight that lunch GL specifically contributes to worsening plasma Aβ biomarkers. This result is unexpected because lunch is the main meal of the day in France and contains large amounts of vegetables and legumes (i.e., fibers), fat, and protein [10], which contribute to limiting the increase in blood glucose [21]. Thus, for this meal, GL might reflect the postprandial glycemia and insulin demand to a lesser extent [22]. Moreover, 47% of individuals in the high-daily-GL tertile group had a good adherence to the Mediterranean diet (score from 6 to 9), and healthy meals (i.e., composed of vegetables, fruits and some cereals) have been associated with lower Aβ burden [20]. However, two plausible explanations can be suggested. First, as lunch is the main meal of the day in France, with various food types, lunch GL could be a good proxy of GL inter-individual variability, although lunch GL is not the best proxy of postprandial glycemia. In our sample, the GL of the other meals might not have been diverse enough to capture information, and the daily GL may have smoothed out this low variability by summing the different GL values. The second explanation concerns the lunch composition. An increase in lunch GL might be associated with a decrease in the consumption of fat and protein that could be protective factors. Indeed, it has been shown that replacing carbohydrates with fat decreases fasting triglycerides and glycemia and increases HDL cholesterol [33,34]. Particularly, the increased consumption of plant-based proteins and fat instead of carbohydrates has been associated with a significant decrease in all-cause mortality [35]. Moreover, if fewer proteins and fat are consumed, GL could ultimately be a good proxy of postprandial glycemia and insulin demand.

Our results suggest that high-GL diets could negatively affect plasma Aβ biomarkers. A possible biological explanation is that high GL indirectly participates in the mechanisms involving Aβ burden. First, a high-GL diet could promote insulin resistance through inflammation and oxidative stress [36,37]. This increases the blood-brain barrier permeability, and consequently, Aβ transport and clearance are impaired [38]. Second, Aβ degradation by an insulin-degrading enzyme (IDE) could be inhibited by GL-induced hyperinsulinemia. Both insulin and Aβ are IDE ligands, and excess insulin could compete with Aβ and, consequently, decrease its clearance [39]. Moreover, it has been shown that hyperinsulinemia modulates plasma Aβ peptide concentration in people with normal cognition [40,41]. However, moving to a healthy diet rich in anti-oxidants and anti-inflammatory components that improve insulin sensitivity could compensate for the harmful effect of a high-GL diet [42,43].

In this study, we did not find an interactive effect of APOE4 status. Yet, higher amyloid deposits were detected in the brain of APOE4 carriers with AD or with normal cognition [44,45]. Moreover, ApoE4 promotes blood–brain barrier disruption, increases Aβ aggregation, and decreases Aβ clearance [46]. Our study may have lacked the statistical power to show a significant interaction between GL and APOE4 carrier status.

This study presents some limitations. First, Aβ peptide concentration was measured in the early 2000s. Since then, more sensitive and effective methods have been developed [47,48]. Moreover, these results could not be compared with Aβ in cerebrospinal fluid because it was not measured in the 3C study. Second, our results concern a single Aβ measurement in plasma, but possible confounding factors were taken into account in the analysis. Specifically, our results are independent from serum creatinine, total cholesterol, and diet quality, three factors that could influence Aβ concentration in plasma [20,26,27], even though these factors cannot truly take into account the lipid dyshomeostasis that could interfere with Aβ trafficking [49]. Third, we assumed that GL did not change between baseline and the time when FFQ was completed. However, the diets measured with the FFQ might not reflect the participants’ diets at baseline. Although this is a cross-sectional analysis, the prospective population-based design with a long follow-up period allowed us to consider reverse causation concerning dementia in the 15 years of follow-up.

## 5. Conclusions

Dietary GL is associated with plasma Aβ_42_ concentration and Aβ_42_/Aβ_40_ ratio independently of the APOE4 carrier status, suggesting that AD biomarkers can be modulated by diet. As AD biomarker changes can be detected a long time before cognitive symptom appearance, these findings support the idea that long-term dietary choices constitute a critical environmental factor in the AD causal pathway. These findings pave the way for identifying new therapeutic targets and preventive measures because diet is a potentially modifiable risk factor. Experimental studies are now required to determine the underlying mechanism linking refined carbohydrate consumption and plasma Aβ peptides with dementia development.

## Figures and Tables

**Table 1:** Characteristics of the study sample according to the tertile of the total GL residuals.

Characteristics	All Sample	Low (<−5.8)	Middle (−5.8 to 5.2)	High (≥5.2)
Sample size, *n*	377	126	125	126
Montpellier center, *n* (%)	204 (54.1)	71 (56.3)	70 (56.0)	63 (50.0)
Age, mean (SD), years	76.1 (5.2)	76.7 (5.7)	75.5 (4.7)	76.2 (5.0)
Women, *n* (%)	227 (60.2)	85 (67.5)	73 (58.4)	69 (54.8)
Education level, *n* (%) ^1^				
No school	104 (27.6)	32 (25.4)	35 (28.0)	37 (29.7)
Primary school	101 (26.8)	37 (29.4)	38 (30.4)	26 (20.6)
High school	88 (23.3)	30 (23.8)	25 (20.0)	33 (26.2)
Graduated	83 (22.0)	27 (21.4)	27 (21.6)	29 (23.0)
APOE4 carriers, *n* (%)	68 (18.0)	22 (17.5)	26 (20.8)	20 (15.9)
Creatinine, mean (SD), mmol/L	82.0 (18.7)	79.7 (19.1)	82.9 (17.6)	83.5 (19.3)
Total cholesterol, mean (SD), mmol/L	5.92 (1.0)	5.88 (0.90)	5.95 (0.97)	5.91 (1.13)
Mediterranean-like diet, *n*. (%) ^1^				
0–3	64 (17.0)	31 (24.6)	18 (14.9)	15 (11.5)
4–5	141 (37.4)	51 (40.5)	51 (40.8)	41 (31.5)
6–9	142 (37.7)	34 (27.0)	47 (38.8)	61 (46.9)
Missing values	30 (7.96)	10 (7.94)	9 (7.20)	11 (8.73)
Energy intake, mean (SD), kcal/day	1193 (378)	1210 (444)	1196 (368)	1173 (312)
Plasma Aβ_40_, mean (SD), pg/mL	231 (81.1)	227 (45.6)	222 (55.0)	245 (120)
Plasma Aβ_42_, mean (SD), pg/mL	39.5 (13.5)	39.1 (10.2)	38.0 (9.92)	41.2 (18.6)
Plasma Aβ_42_/Aβ_40_ ratio, mean (SD)	0.176 (0.046)	0.176 (0.043)	0.178 (0.055)	0.174 (0.04)

Abbreviation: Aβ, amyloid-β; APOE4, apolipoprotein e ε4 allele; SD, standard deviation. ^1^ Missing data: education level, 0.27%; Mediterranean-like diet, 8%.

**Table 2:** Association between glycemic load residuals and amyloid-β peptides.

Glycemic Load Residuals	Aβ_40_ *n* = 372		Aβ_42_ *n* = 373		Aβ_42_/Aβ_40_ *n* = 374	
	β (CI)	*p* Value	β (CI)	*p* Value	β (CI)	*p* Value
Model 1						
Daily	1.72 (−2.24, 5.68)	0.394	0.04 (−0.73, 0.81)	0.9222	−0.0014 (−0.0044, 0.0016)	0.3607
Breakfast	6.77 (−1.08, 14.6)	0.0906	0.76 (−0.77, 2.29)	0.3298	−0.0011 (−0.0072, 0.005)	0.72
Lunch	2.82 (−7.89, 13.5)	0.6047	−1.97 (−4.04, 0.11)	0.0631	−0.0094 (−0.0176, −0.0012)	**0.024**
Afternoon snack	3.83 (−9.83, 17.5)	0.5815	0.17 (−2.5, 2.84)	0.8993	−0.0031 (−0.0137, 0.0076)	0.574
Dinner	0.27 (−9.42, 9.96)	0.9566	0.05 (−1.83, 1.94)	0.9561	−0.0025 (−0.01, 0.005)	0.5094
Model 2						
Daily	0.61 (−3.39, 4.62)	0.7632	−0.02 (−0.80, 0.76)	0.9648	−0.0011 (−0.0042, 0.002)	0.5037
Breakfast	4.79 (−3.22, 12.8)	0.2403	0.7 (−0.86, 2.27)	0.3792	−0.0004 (−0.0067, 0.0059)	0.9012
Lunch	0.66 (−10.0, 11.3)	0.9026	−2.2 (−4.27, −0.12)	**0.0378**	−0.0089 (−0.0172, −0.0007)	**0.0344**
Afternoon snack	1.08 (−12.5, 14.7)	0.8761	−0.26 (−2.92, 2.41)	0.8504	−0.003 (−0.0138, 0.0078)	0.5851
Dinner	−0.53 (−10.1, 9.07)	0.9131	0 (−1.87, 1.88)	0.9971	−0.0022 (−0.0097, 0.0053)	0.5628

Abbreviation: Aβ, amyloid-β; CI, confidence interval; GL, glycemic load. Model 1 was adjusted for center, age, sex, education level, APOE4 status, and energy intake. Model 2 was additionally adjusted for serum creatinine, total cholesterol, and Mediterranean-like diet. β for a 10-point increase in the GL value per day (equivalent to eating an additional 30 g of a French baguette at each corresponding meal). *p* < 0.05 are in bold.

## Data Availability

Data described in the manuscript, code book, and analytic code will be made available upon request to the 3C scientific committee.

## Supplementary Materials

The following supporting information can be downloaded at: https://www.mdpi.com/article/10.3390/nu14122485/s1, Table S1: Glycemic load values of each item of the FFQ and mean glycemic load of the sample according to the meal types; Table S2: Characteristics of the study sample; Table S3: Association between plasma amyloid-β peptides and glycemic load residuals after exclusion of participants with incident dementia (*n* = 51).
