# Supplementary material for: Dietary Glycemic Load and Plasma Amyloid-β Biomarkers of Alzheimer’s Disease
Source: Nutrients. 2022 Jun 15;14(12):2485. doi: 10.3390/nu14122485 (PMC9230608; doi:10.3390/nu14122485)
Supplement: Supplementary file 1 [file nutrients-14-02485-s001.zip › nutrients-1701753-supplementary.pdf]

Supplementary Files

Table S1. Glycemic load values of each item of the FFQ and mean glycemic load of the sample according to the meal types.

**FFQ items**	**Glycemic load values**			
	Breakfast	Lunch	Afternoon-snack	Dinner
Sandwich		13.5		13.5
Pasta		29		29
Rice		23		23
Potatoes		17		17
Legumes		5		5
Pizza		11		6.24
Soup		11	11	11
Bread, baguette	13.5	13.5	13.5	13.5
Rusk	7.8		7.8	
Jam (or honey)	10.2		10.2	
Pastries	15.34		15.34	
Cereal	20.25		20.25	
Biscuits	13.44		13.44	
Cakes	13.44		13.44	
Fruits	6.24	6.24	6.24	6.24
Sugar	6.5		6.5	
Sweets, chocolate, chocolate bars			21.84	
Stewed fruit	8.17	8.17	8.17	8.17
Yogurt	2.66	2.66	2.66	2.66
Milk	3.9	3.9	3.9	3.9
Hot chocolate	12.04		12.04	
Fruit juice	16.38	16.38	16.38	16.38
Soda	16.38	16.38	16.38	16.38
Beer	5.28	5.28	5.28	5.28
**Mean (SD) range**	25.8 (12.7) 0 – 67	35.8 (11.8) 0 – 96	8.4 (10.7) 0 – 61	34.1 (13.3) 0 – 85

Table S2. Characteristics of the study sample.

Characteristics	Whole sample	Dementia-free	Incident dementia
Sample size, *n*	377	326	51
Montpellier center, *n* (%)	204 (54.1)	186 (57.0)	18 (35.3)
Age, mean (SD), years	76.1 (5.2)	76.0 (5.2)	76.9 (4.8)
Women, *n* (%)	227 (60.2)	192 (58.9)	35 (68.6)
Education level, *n* (%) [1]			
No school	104 (27.6)	83 (25.5)	21 (41.2)
Primary school	101 (26.8)	86 (26.4)	15 (29.4)
High school	88 (23.3)	79 (24.2)	9 (17.6)
Graduate	83 (22.0)	77 (23.6)	6 (11.8)
APOE4 carriers, *n* (%)	68 (18.0)	55 (16.9)	13 (25.5)
Creatinine, mean (SD), mmol/L	82.0 (18.7)	82.0 (18.9)	81.9 (17.4)
Total cholesterol, mean (SD), mmol/L	5.92 (1.0)	5.92 (1.01)	5.87 (0.97)
Mediterranean-like diet, *n* (%) 1			
0-3	64 (17.0)	52 (16.0)	12 (23.5)
4-5	141 (37.4)	121 (37.1)	20 (39.2)
6-9	142 (37.7)	127 (39.0)	15 (29.4)
Missing values	30 (7.96)	26 (7.98)	4 (7.84)
Energy intake, mean (SD), kcal/day	1193 (378)	1205 (387)	1115 (306)
Glycemic load, mean (SD), /day	111.6 (35.5)	113.0 (36.0)	102.6 (30.7)
Glycemic load residuals, mean (SD)	0 (13.7)	0.347 (13.7)	-2.22 (13.4)
Plasma $A\beta_{40}$, mean (SD), pg/mL	231.1 (81.1)	232.1 (84.6)	225.1 (53.5)
Plasma $A\beta_{42}$, mean (SD), pg/mL	39.50 (13.5)	39.33 (14.1)	40.28 (9.0)
Plasma $A\beta_{42}/A\beta_{40}$, mean (SD)	0.176 (0.046)	0.175 (0.046)	0.186 (0.049)

Abbreviation: Aβ. amyloid-β; APOE4. Apolipoprotein e ε4 allele; SD. standard deviation.
[1] Missing data: education level. 0.27%; Mediterranean-like diet. 8%.

Table S3. Association between plasma amyloid-β peptides and glycemic load residuals after exclusion of participants with incident dementia (n = 51).

Glycemic load residuals	$A\beta_{40}$ n = 321		$A\beta_{42}$ n = 323		$A\beta_{42}/A\beta_{40}$ n = 323	
	β (CI)	*P* value	β (CI)	*P* value	β (CI)	*P* value
Daily	0.53 (-3.75. 4.8)	0.8091	-0.09 (-0.93. 0.76)	0.8407	-0.0014 (-0.0046. 0.0017)	0.375
Breakfast	4.4 (-4.21. 13.0)	0.3153	0.81 (-0.90. 2.52)	0.3538	0.0005 (-0.006. 0.007)	0.8865
Lunch	1.58 (-9.98. 13.1)	0.7881	-2.56 (-4.84. -0.27)	**0.0282**	-0.0124 (-0.0213. -0.0036)	**0.0058**
Afternoon snack	1.33 (-12.9. 15.6)	0.8542	-0.29 (-3.14. 2.56)	0.8431	-0.0035 (-0.0145. 0.0074)	0.5255
Dinner	-1.04 (-11.3. 9.21)	0.8414	-0.1 (-2.14. 1.93)	0.9204	-0.0023 (-0.01. 0.0055)	0.5648

Abbreviation: Aβ. amyloid-β; CI. confidence interval; GL. glycemic load.

Model was adjusted for center. age. sex. education level. APOE4. energy intake. serum creatinine. total cholesterol. and Mediterranean-like diet.

β value for a 10-point increase in the GL value per day (equivalent to eating an additional 30g of a French baguette at each corresponding meal). $p < 0.05$ are in bold.
